# Expression of schizophrenia biomarkers in extraocular muscles from patients with strabismus: an explanation for the link between exotropia and schizophrenia?

**DOI:** 10.7717/peerj.4214

**Published:** 2017-12-22

**Authors:** Andrea B. Agarwal, Austin J. Christensen, Cheng-Yuan Feng, Dan Wen, L. Alan Johnson, Christopher S. von Bartheld

**Affiliations:** 1 Department of Physiology and Cell Biology, University of Nevada, Reno School of Medicine, Reno, NV, USA; 2 Department of Ophthalmology, Xiangya Hospital, Central South University, Changsha, China; 3 Sierra Eye Associates, Reno, NV, USA

**Keywords:** Strabismus, Gene expression, PCR Array, Growth factor, Extraocular muscle, Biomarker, Extracellular matrix, Schizophrenia

## Abstract

Recent studies have implicated exotropia as a risk factor for schizophrenia. We determined whether schizophrenia biomarkers have abnormal levels of expression in extraocular muscles from patients with strabismus and explored whether differences in gene expression between medial and lateral rectus muscles may explain the specific association of schizophrenia with exotropia but not esotropia. Samples from horizontal extraocular muscles were obtained during strabismus surgery and compared with age- and muscle type-matched normal muscles from organ donors. We used PCR arrays to identify differences in gene expression among 417 signaling molecules. We then focused on established schizophrenia-related growth factors, cytokines, and regulators of the extracellular matrix. Among 36 genes with significantly altered gene expression in dysfunctional horizontal rectus muscles, over one third were schizophrenia-related: CTGF, CXCR4, IL1B, IL10RA, MIF, MMP2, NPY1R, NRG1, NTRK2, SERPINA3, TIMP1, TIMP2, and TNF (adjusted *p* value ≤ 0.016667). By PCR array, expression of three of these genes was significantly different in medial rectus muscles, while eleven were significantly altered in lateral rectus muscles. Comparing baseline levels between muscle types, three schizophrenia-related genes (NPY1R, NTRK2, TIMP2) had lower levels of expression in medial rectus muscles. Despite the surprisingly large number of schizophrenia-related genes with altered gene expression levels in dysfunctional muscles, the lack of specificity for medial rectus muscles undermines a model of shared, region-specific gene expression abnormalities between exotropia and schizophrenia, but rather suggests consideration of the alternative model: that exotropia-induced aberrant early visual experiences may enable and/or contribute as a causative factor to the development of schizophrenia.

## Introduction

Studies conducted in the last decade have shown that people with exotropia are more likely to develop schizophrenia than the general population (Korn, 2004; Toyota et al., 2004; Schiffman et al., 2006; Yoshitsugu et al., 2006; Ndlovu et al., 2011; Toyosima et al., 2011). Curiously, schizophrenia is linked specifically to exotropia, but not (or much less) to esotropia, with the risk of schizophrenia being most strikingly increased in children with constant exotropia (Toyota et al., 2004; Yoshitsugu et al., 2006; Ndlovu et al., 2011). Co-occurrence of exotropia and psychosis has been noticed previously (Hinton, 1963; Leskowitz, 1984; Krakauer et al., 1995). The reason for the specific association between exotropia and schizophrenia has remained enigmatic (Toyota et al., 2004; Ndlovu et al., 2011), although theories have been proposed (Korn, 2004).

Recent work has indicated that many cytokines, growth factors and regulators of the extracellular matrix involved in early brain development also control plasticity and adaptation of skeletal muscles, including extraocular muscles (EOMs) (Altick et al., 2012; Stager Jr, McLoon & Felius, 2013; Mienaltowski & Birk, 2014; Agarwal et al., 2016). Levels of expression of such genes are altered in strabismic EOMs (Christensen et al., 2015; Von Bartheld et al., 2016; Agarwal et al., 2016). A large fraction of genes expressed in EOMs, as opposed to limb muscles, is devoted to neural signaling (Fischer et al., 2005). Taken together, this raises the possibility that some of the genes regulating EOM development and plasticity may be schizophrenia-related. So far, only a few risk genes were shown to be shared between strabismus and schizophrenia: PMX2B or PHOX2B/ARIX (Toyota et al., 2004; Toyosima et al., 2011), and AHI1 (Torri et al., 2010; Min et al., 2017), although additional ones may exist. Furthermore, several loci on chromosomes have been implicated in both strabismus and schizophrenia, e.g., 7q31.2 (Shaaban et al., 2009; Bassett et al., 2011), and 22q11.2 (Karayiorgou et al., 1995; Faraone et al., 1998; Toyosima et al., 2011).

When misalignments of the visual axis are corrected surgically, one procedure, called a resection, is performed on muscles that require “strengthening,” and in such cases a portion of the muscle and/or its tendon becomes available for analysis. In people with exotropia, the medial rectus muscle is typically resected, while in esotropia, the lateral rectus muscle is resected to restore alignment (Von Noorden & Campos, 2002).

**Figure 1:**
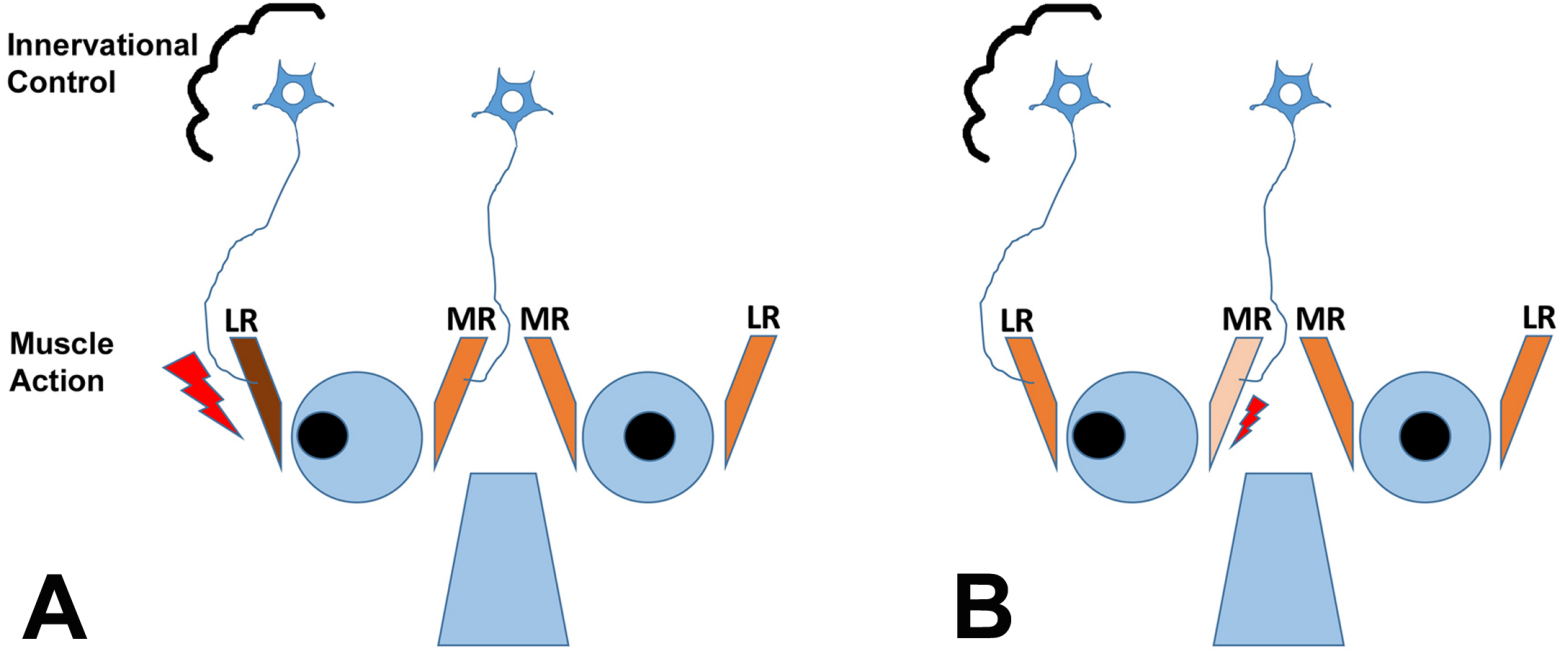
The cartoon depicts two scenarios (A and B) for a patient with exotropia, indicating muscle action of the agonist/antagonist horizontal eye muscles: the medial (MR) and lateral (LR) rectus muscles. (A) shows one possibility, that the LR is overactive (too strong, large lightning bolt); (B) shows another possibility, that the MR is underacting (too weak, small lightning bolt). The two possibilities are not mutually exclusive. Recent work indicates that the agonist can be rebuilt in horizontal strabismus and that rebuilding at an appropriate muscle length aligns the eyes, indicating that properties of the agonist in strabismus are mechanistically involved (Debert et al., 2016). The cartoon also shows the second possible site of impairment, the innervational control, which is thought to also cause strabismus (for a detailed discussion of the causes of strabismus, see Von Noorden & Campos, 2002; Von Bartheld, Croes & Johnson, 2010).

It is important to understand that theoretically, horizontal strabismus may arise because the agonist muscle is too weak, the antagonist muscle is too strong, or a combination of the two conditions exists, as illustrated for exotropia in Fig. 1. The finding that regeneration of the agonist muscle after injection of a myotoxin, without any direct manipulation of the antagonist, can significantly reduce the misalignment (Debert et al., 2016), indicates that the agonist (medial rectus muscle in exotropia, lateral rectus muscle in esotropia) is indeed dysfunctional, although contributions of the antagonist may exist as well. It is also important to emphasize that changes in gene expression in EOMs from strabismic people do not imply that the etiology of the strabismus necessarily derives from the muscle itself, since changes in gene expression may be in response to altered demand or in response to innervational imbalance (Von Noorden & Campos, 2002; Lennerstrand, 2007). Most theories on the etiology of strabismus consider a combination of mechanical and innervational factors (reviewed by Von Noorden & Campos, 2002). For the purposes of the current study, we are not making any assumptions about primary causes of the strabismus. We do make the assumption that the agonist (that is available for analysis) is substantively involved in deviations, based on recent evidence (Debert et al., 2016).

While changes in gene expression may be expected to be similar between the medial and lateral rectus muscle from people with horizontal strabismus (Agarwal et al., 2016), this is not necessarily the case: these two muscles derive embryonically from different tissues (Noden & Trainor, 2005), and are controlled by different transcription factors and signaling pathways (Nakano et al., 2001; Noden & Trainor, 2005; Lin et al., 2006). The two rectus muscles are innervated by different nerves: the oculomotor nerve from the midbrain for the medial rectus, and the abducens nerve from the hindbrain for the lateral rectus. By analyzing resected muscle samples, it is possible to determine whether the presumed structural asymmetries between agonist and antagonist horizontal rectus muscles (Scott, 1994; Kraft, 2017) have a correlate at the gene expression level, and to find out whether schizophrenia-relevant gene expression changes are more common in medial rectus muscles than lateral rectus muscles.

**Figure 2:**
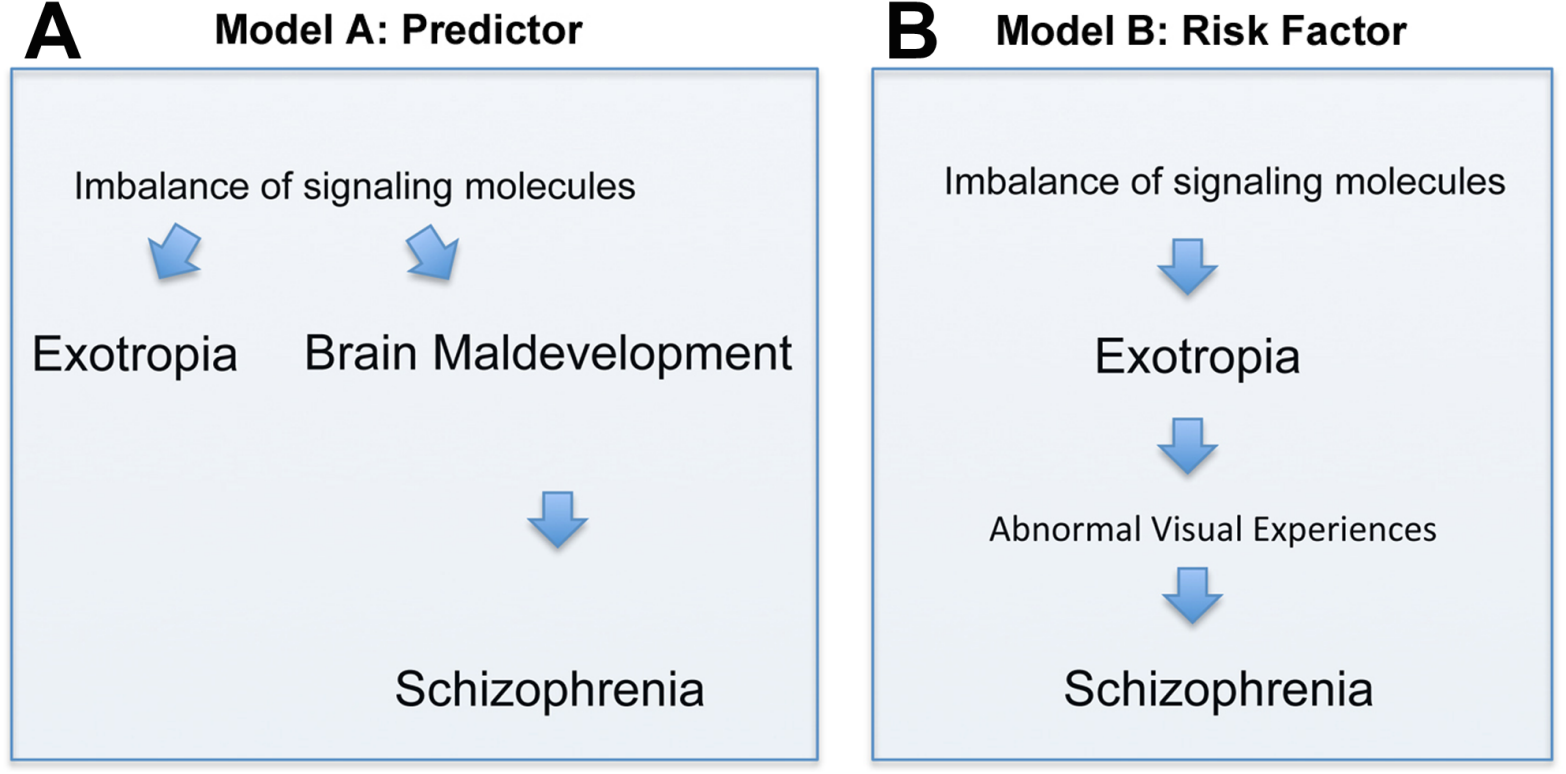
Depiction of two models (Models A and B) of how the correlation between exotropia and schizophrenia may be interpreted, keeping in mind that correlation alone is not evidence for causation. In both models, imbalances of signaling molecules are considered essential. (A) In model A, exotropia is an epiphenomenon, not causally related to schizophrenia, but a predictor. (B) In model B, exotropia is—possibly—causally involved in schizophrenia, by inducing abnormal early visual experiences, and therefore may be a true risk factor.

There are two principal interpretations of the association between exotropia and schizophrenia. Exotropia, which typically presents much earlier in life than schizophrenia, may be an epiphenomenon that does not causally influence the development of schizophrenia (although it may be a predictor, Fig. 2A). Alternatively, exotropia may contribute as a causal factor to the development of schizophrenia, being a true risk factor, possibly because exotropia induces abnormal early visual experiences that contribute to the development of schizophrenia (Fig. 2B). In the first scenario, one would expect the medial rectus muscle from people with exotropia to have a more divergent pattern of gene expression than the lateral rectus muscle from people with esotropia. Differences or similarities between medial and lateral rectus muscles in terms of altered expression levels of schizophrenia-related genes thus may help to favor one over the other model. In our study, we asked whether signaling molecules, known to be risk factors and/or biomarkers for schizophrenia, have altered gene expression in EOMs from people with strabismus, and whether any such imbalances of gene expression are more common in the medial rectus than the lateral rectus muscle. Even though we cannot provide evidence that exotropia is causative to schizophrenia, we can show that the model postulating causation *cannot be ruled out*. To our knowledge, the current study is the first attempt to define a selection of genes with imbalanced expression in EOMs that may be relevant for schizophrenia.

## Materials & Methods

### Sources of tissues

Human extraocular muscle (EOM) samples were obtained during strabismus correction surgery and from deceased organ donors. Experimental procedures of human tissues were conducted in compliance with the declaration of Helsinki and conformed to the requirements of the United States Health Insurance Portability and Privacy Act. All patients gave written consent, and the institutional review board (IRB) of the University of Nevada, Reno approved the research involving human subjects (509109-9). Samples consisted of distal segments of horizontal rectus muscles (including the myotendinous junction). Medial rectus samples were obtained from patients with exotropia, and lateralrectus samples from patients with esotropia (for demographic information, see Tables S1 and S2), because these respective muscles are typically resected in order to “strengthen” them (Von Noorden & Campos, 2002), while recessed muscles typically generate no or very little muscle tissue for analysis. Nearly all samples were taken from muscles that appeared to have normal contractility (were not overtly overacting or underacting), although we did not perform any detailed or quantitative analysis of contractile force. No data were obtained for the muscle antagonist that was not resected (e.g., the lateral rectus in case of a patient with exotropia), because such tissues do not become available at all, or not in sufficient quantities, during strabismus surgeries. Special care was taken to ensure that samples contained similar amounts of muscle vs. tendon, to prevent comparison of muscle-rich samples with tendon-rich samples. We chose to examine gene expression (quantitative PCR) for our study, because this approach proved to be significantly more sensitive than protein expression by proteomics analyses (Agarwal et al., 2016).

### Muscle samples

Samples were collected either during surgery or, in case of normal EOMs, from organ donors within 1–4 h of death. Tissue samples were immersed in Allprotect (#76405; Qiagen, Valencia, CA, USA) or in RNAlater (#AM7022; Ambion, Austin, TX, USA) and stored at −80 °C until processed. Samples from organ donors with a history of strabismus, eye surgery, muscle disease, or neurological disease were excluded from further analysis. A total of 190 samples (154 from patients with strabismus and 36 normal) were collected for gene expression analysis; among these samples, 56 were selected for PCR arrays and paired by EOM type, RNA quality, muscle/tendon content, and age at surgery. Such age-matching was designed to eliminate or reduce any fluctuations in gene expression due to normal aging. PCR array samples were from patients with a mean age of 23.7 years (range: 2–80, ratios of male/female/gender unknown: 21/24/1), and from donors with a mean age of 18.8 years (range: 6–38 years, male/female ratio: 7/3) (for details, see Table S3).

### Cross-comparison experimental schemes

To detect possible differences in gene expression, we compared relative expression levels in the following muscle pairings: normal medial rectus vs. medial rectus from patients with exotropia; normal lateral rectus vs. lateral rectus from patients with esotropia; normal medial rectus vs. normal lateral rectus; medial rectus from patients with exotropia vs. lateral rectus from patients with esotropia. These pairings were chosen to examine the possibility that expression levels are different between the two types of muscle in normal conditions, or only in strabismic conditions, which would be missed in the simple comparisons of “before and after” pairs = normal vs. disease conditions for the same muscle type. For details and the original data tables, see Tables S4–S8.

### RNA collection

Muscle samples were thawed, weighed, wrapped in foil, pulverized in liquid nitrogen, transferred into chilled TRIzol (#15596-026; Life Technologies, Grand Island, NY, USA), homogenized, and centrifuged. Chloroform was added to the supernatant, tubes shaken vigorously, and centrifuged. Supernatant was poured into microcentrifuge tubes, ethanol added, vortexed, and loaded onto columns for RNA isolation (RNeasy Lipid Tissue Mini Kit, #74804; Qiagen, Valencia, CA, USA). The manufacturer’s kit protocol for total RNA isolation was followed, including on-column DNase digestion. After analysis of quantity and quality on an Agilent 2100 BioAnalyzer, RNA samples were stored at −80°C until used for reverse transcription and PCR array.

### Reverse transcription and PCR arrays

Reverse transcription was performed using RT^2^ First Strand Kit (#330401; SABiosciences, Frederick, MD, USA), as previously described (Agarwal et al., 2016). In brief, similar amounts of RNA (750–880 ng) were added to each reverse transcriptase reaction in order to produce similar amounts of cDNA. The cDNA was used for PCR array immediately or after storage for 2–3 h at −20°C. All experiments were conducted as pairs, with samples containing cDNA from between one and four muscles for each sample in a pair. Once the large fraction of schizophrenia-related genes had become apparent, we specifically targeted additional schizophrenia-related genes in two custom PCR arrays. Expression of a total of 417 different genes was examined; 37 of these were from two custom PCR arrays (SABiosciences), and another 380 different genes were examined on five types of Human SABiosciences arrays: Common Cytokines (PAHS-021ZC), Neurotrophins and Receptors (PAHS-031ZC), Tyrosine Kinases (PAHS-161ZC), Neurogenesis (PAHS-404ZC), and Myogenesis/Myopathy (PAHS-099ZC), according to the manufacturer’s protocol with SYBR Green/ROX quantitative PCR (qPCR) Master Mix (SABiosciences 330521). All arrays were processed on Applied Biosystems 7900HT real-time PCR Systems. Data were collected using SDS 2.4 software, applying the same baseline and threshold values for all samples.

### Data analysis for PCR arrays

Data files were exported from SDS 2.4 and analyzed using web-based SABiosciences software (http://www.sabiosciences.com/pcrarraydataanalysis.php) which calculates differences in relative gene expression using the ΔΔCt method. We normalized the expression data to reference genes that were most consistently expressed in the array plates, usually ACTB, GAPDH, and RPLP0. We compiled all genes that were up- or down-regulated twofold or more, and below or near the adjusted *p*-value for the experiment, as shown for 59 different genes in the Tables. The *p*-values were calculated based on a Student’s *t*-test of the replicate 2(^−ΔC*t*^) values for each gene in the donor vs. strabismic patient groups, or donor vs. donor groups, or patients with exotropia vs. patients with esotropia groups. All comparisons were conducted as separate pairs, so that no individual array results were used in more than one comparison, making the Student’s *t*-test the appropriate tool for statistical analysis (see Tables S4–S8). To account for multiple comparisons and to control the false discovery rate, we calculated an adjusted *p*-value for each experiment as detailed in the legends for Tables, using the method of Benjamini & Hochberg (1995), and only those genes with p-values that were below the *adjusted p*-value were ultimately considered to be significantly altered in their expression.

### Selection of schizophrenia-relevant genes

To identify proteins or genes that are considered established schizophrenia biomarkers or risk factors, we consulted recent meta-analyses, reviews and primary literature based on quantification of proteins in blood or cerebrospinal fluid, or gene and protein expression in brain tissues, from patients with schizophrenia (Carter, 2009; Schwarz et al., 2010; Miller et al., 2011; Ayalew et al., 2012; Berretta, 2012; Pandya, Kutiyanawalla & Pillai, 2013). Relevant references, with evidence for the gene being related to schizophrenia, are provided in each of the Tables presenting the data.

## Results

### Many signaling molecules with altered gene expression in horizontal rectus muscles from patients with strabismus were schizophrenia-related

For convenience, we have compiled in Table 1 the full names of all genes mentioned in the text, figures or tables, listed alphabetically by the gene abbreviation. First, we used PCR arrays to survey 396 genes encoding signaling molecules, and found that among these genes, 36 had significantly altered levels of expression (based on adjusted *p*-values according to Benjamini & Hochberg, 1995) in the muscles from patients with strabismus compared to normal control muscles. Of these 36 genes, a surprisingly large number (13 = 36.1%) were schizophrenia-related biomarkers or risk genes (Table 2). The schizophrenia-related genes included two that were down-regulated, NRG1 and SERPINA3, and 11 that were up-regulated: CTGF, CXCR4, IL1B, IL10RA, MIF, MMP2, NPY1R, NTRK2, TIMP1, TIMP2, and TNF (statistically significant, with an adjusted *p* ≤ 0.016667). Overall, this suggested that altered expression of genes in EOMs may inform about the link between strabismus and schizophrenia—either as depicted in model A (epiphenomenon) or model B (possible contribution to causation) (Figs. 2A and 2B). Since our initial dataset (Christensen et al., 2015; Von Bartheld et al., 2016) compiled data for medial and lateral rectus muscles combined, there was no information for muscle specificity. Therefore, we next asked whether any or all of the dysregulated schizophrenia-related genes may be specifically altered within the medial rectus muscle, samples of which are typically obtained from patients with exotropia.

**Table 1:** Genes presented in Text, Tables 2–6, and figures. Genes are listed alphabetically by gene symbol abbreviation.

Gene symbol	Gene name/description
ACTB	Actin beta
ACVR2B	Activin A receptor type 2B
AHI1	Abelson helper integration site 1
AKT1	AKT serine/threonine kinase 1 (protein kinase B)
ATP2A1	ATPase sarcoplasmic/endoplasmic reticulum Ca^2+^ transporting 1 (SERCA1)
BDNF	Brain derived neurotrophic factor
BMP4	Bone morphogenetic protein 4
CAV3	Caveolin 3
CCL16	C-C motif chemokine ligand 16
CNTF	Ciliary neurotrophic factor
CNTFR	Ciliary neurotrophic factor receptor
CNTN1	Contactin 1
CRYAB	Crystallin alpha B
CS	Citrate synthase
CTGF	Connective tissue growth factor
CXCR4	C-X-C motif chemokine receptor 4
DCX	Doublecortin
DDR2	Discoidin domain receptor tyrosine kinase 2
DES	Desmin
DISC1	Disrupted in schizophrenia 1
DMD	Dystrophin
DYSF	Dysferlin
GAPDH	Glyceraldehyde-3-phosphate dehydrogenase
GDF9	Growth differentiation factor 9
GDNF	Glial cell derived neurotrophic factor
GFRA2	GDNF family receptor alpha 2
GSK3B	Glycogen synthase kinase 3
ICAM1	Intercellular adhesion molecule 1
IFNG	Interferon gamma
IGF1	Insulin like growth factor 1
IKBKB	Inhibitor of nuclear factor kappa B kinase subunit beta
IL1A	Interleukin 1 alpha
IL1B	Interleukin 1 beta
IL6R	Interleukin 6 receptor
IL7	Interleukin 7
IL10RA	Interleukin 10 receptor subunit alpha
MIF	Macrophage migration inhibitory factor
MMP1	Matrix metallopeptidase 1
MMP2	Matrix metallopeptidase 2
MMP9	Matrix metallopeptidase 9
MUSK	Muscle associated receptor tyrosine kinase
MYH2	Myosin heavy chain 2
NEB	Nebulin
NGF	Nerve growth factor
NLGN1	Neuroligin 1
NOTCH2	Notch 2
NPFFR2	Neuropeptide FF receptor 2
NPY1R	Neuropeptide Y receptor Y1
NRCAM	Neuronal cell adhesion molecule
NRG1	Neuregulin 1
NT-4	Neurotrophin 4
NTRK1	Neurotrophic receptor tyrosine kinase 1
NTRK2	Neurotrophic receptor tyrosine kinase 2
PAX3	Paired box 3
PAX6	Paired box 6
PAX7	Paired box 7
PMX2B/PHOX2B	Paired like homeobox 2b
PPARGC1A	Peroxisome proliferator-activated receptor gamma coactivator 1 alpha
PPARGC1B	Peroxisome proliferator-activated receptor gamma coactivator 1 beta
PRKAB2	Protein kinase AMP-activated non-catalytic subunit beta 2
PRKAG3	Protein kinase AMP-activated non-catalytic subunit gamma 2
PTGER2	Prostaglandin E receptor 2
RELN	Reelin
RPLP0	Ribosomal protein lateral stalk subunit P0
SERPINA3	Serpin family A member 3
SLC2A4	Solute carrier family 2 member 4
SLIT2	Slit guidance ligand 2
SPP1	Secreted phosphoprotein 1
TCF4	Transcription factor 4
TGFB1	Transforming growth factor beta 1
TIMP1	Tissue inhibitor of metalloproteinases 1
TIMP2	Tissue inhibitor of metalloproteinases 2
TNF	Tumor necrosis factor
TNNI2	Troponin I2, fast skeletal type
TNNT3	Troponin T3, fast skeletal type
TRIM63	Tripartite motif containing 63
TTN	Titin

**Table 2:** Altered expression of signaling molecules in horizontal rectus muscles from patients with strabismus, with gray shading indicating schizophrenia-related genes. The fold-difference compared with normal (donor) horizontal rectus muscles is shown. For gene names, see Table 1.

	Fold difference					
Gene symbol	Increase +	Decrease −	*n*	*p*-value	Confirmation	Reference for schizophrenia
**ATP2A1**		0.14	5	**0.008312**		
**CCL16**	7.80		5	**0.001982**		
**CNTN1**	11.06		5	**0.002932**	MA 5.39	
**CRYAB**		0.30	5	**0.011055**		
**CS**		0.28	5	**0.000735**		
**CTGF**	6.39		5	**0.009380**	MA 7.02	Schwarz et al. (2010)
**CXCR4**	2.86		8	**0.000256**	MA 5.15	Volk et al. (2015)
DCX	9.59		7	0.020369		
**DDR2**	3.11		7	**0.000991**	MA 3.49	
**GDF9**		0.36	7	**0.014974**		
**GDNF**		0.11	10	**0.002096**	MA 0.40	
**ICAM1**	2.05		5	**0.006826**		
**IKBKB**		0.51	5	**0.012782**	MA 1.98	
**IL1A**	6.64		7	**0.016618**		
**IL1B**	3.70		15	**0.016146**		Miller et al. (2011)
IL7	2.37		7	0.018221	MA 3.78	Schwarz et al. (2010)
**IL10RA**	4.42		8	**0.014755**	MA 3.19	Carter (2009)
**MIF**	1.81		5	**0.005159**		Schwarz et al. (2010)
**MMP2**	4.63		5	**0.000207**	MA 3.68	Schwarz et al. (2010)
NOTCH2	4.96		7	0.027403	MA 3.32	
**NPFFR2**	44.43		8	**0.000138**	MA 8.89	
**NPY1R**	4.51		8	**0.000009**		Vawter et al. (2004)
**NRG1**		0.25	10	**0.000198**		Carter (2009), Ayalew et al. (2012) and Berretta (2012)
**NTRK1**	3.09		10	**0.005728**		
**NTRK2**	3.71		10	**0.004338**	MA 3.34	Pandya, Kutiyanawalla & Pillai (2013)
**PAX3**		0.29	12	**0.015145**		
PAX6	24.85		7	0.020369		
**PAX7**		0.11	10	**0.004492**		
**PPARGC1A**		0.26	4	**0.004602**	MA 0.73	
**PPARGC1B**		0.22	4	**0.001386**		
**PRKAB2**		0.27	5	**0.002002**		
**PRKAG3**		0.27	5	**0.002847**		
**PTGER2**	25.73		8	**0.000195**		
**SERPINA3**		0.23	5	**0.001156**	MA 0.26	Kim et al. (2016) and Tomasik et al. (2016)
**SLC2A4**		0.18	5	**0.001735**		
**SLIT2**	3.51		7	**0.000215**	MA 3.15	
**SPP1**		0.25	7	**0.005122**		
**TIMP1**	7.43		5	**0.000459**	MA 2.87	Schwarz et al. (2010)
**TIMP2**	5.81		5	**0.002852**	MA 3.61	Kumarasinghe et al. (2013)
**TNF**	3.41		12	**0.000083**		Carter (2009) and Miller et al. (2011)

**Notes.**

Genes (symbols listed alphabetically) are included when they were up- or down-regulated 2-fold or more in the horizontal rectus muscle samples from patients with strabismus and had a *p*-value below or near the adjusted p-value for multiple comparisons. We list the fold-difference for the sample obtained from strabismic patients compared with the normal controls, the number of independent samples (*n*), the adjusted p-value (in bold when significant at *p* ≤ 0.0166667 according to Benjamini & Hochberg (1995); those that were near the adjusted p-value are shown in regular font). We also list major references providing evidence when the gene is schizophrenia-related. Genes that are schizophrenia-related (biomarkers or susceptibility genes) are indicated by gray shading. Confirmation of fold-difference (*p* ≤ 0.05) by microarray (MA, Altick et al., 2012) is indicated (for medial rectus muscle). Only genes encoding signaling molecules, but not structural muscle protein (such as DES, DMD, DYSF, MUSK, MYH2, NEB, TNNI2, TNNT3, TRIM63, TTN) are included, since muscle proteins involved in contractility were the focus of our previous publication (Agarwal et al., 2016). Abbreviations used: MA, microarray; n, number of independent experiments (pair-wise comparison of age-matched muscle samples).

### Some of the dysregulated genes in medial rectus muscles from patients with exotropia were schizophrenia-related

When gene expression by PCR arrays was analyzed exclusively in the medial rectus samples, we found that three schizophrenia-related genes were significantly altered: NPY1R, NRG1, and NTRK2 (adjusted *p* ≤ 0.012500, Table 3). NTRK2 was also significantly up-regulated according to our previous microarray analysis (Altick et al., 2012), as indicated in Table 3. Altered expression of another schizophrenia-related gene, TNF, was borderline significant on the PCR arrays, with a *p*-value = 0.013100 (Table 3). Three additional schizophrenia-related genes were not examined by PCR-array, but, based on previous microarray studies, are already known to be significantly up-regulated in medial rectus muscles from patients with exotropia: CTGF, TIMP1 and TIMP2 (Altick et al., 2012). On the other hand, we obtained strong evidence showing that two other schizophrenia-related genes, MMP9 and TGFB1, were *not* significantly altered according to the analysis of our PCR arrays. All of these data for medial rectus muscles from patients with exotropia are compiled in Table 3.

**Table 3:** Altered expression of signaling molecules in *medial* rectus muscles from patients with exotropia, with gray shading indicating schizophrenia-related genes. The fold-difference compared with normal (donor) medial rectus muscles is shown. For gene names, see Table 1.

	Fold difference						
Gene symbol	Increase +	±0	Decrease −	*n*	*p*-value	Confirmation	Reference for schizophrenia
**ACVR2B**			0.35	5	**0.004438**		
**ATP2A1**			0.14	5	**0.008312**		
CAV3			0.35	5	0.034375		
CNTF			0.30	6	0.028100		
**CRYAB**			0.30	5	**0.011055**		
**CS**			0.28	5	**0.000735**		
**CTGF**				(4)	(**0.0369**)	MA 7.02	Schwarz et al. (2010)
CXCR4	2.99			4	0.039900	MA 5.15	Volk et al. (2015)
**GDNF**			0.21	6	**0.002500**	MA 0.40	
**GFRA2**	2.84			3	**0.010870**		
IFNG	2.47			3	0.034400		Müller et al. (2015)
IKBKB			0.51	5	0.012782	MA 1.98	
IL7	3.10			3	0.090100	MA 3.78	Schwarz et al. (2010)
IL10RA	7.17			4	0.089900	MA 3.19	Carter (2009)
**MMP2**				(4)	**(0.0181)**	MA 3.68	Schwarz et al. (2010)
MMP9		1.03		6	0.515200	MA 0.68	Kumarasinghe et al. (2013)
NPFFR2	125.80			4	0.017700	MA 8.89	
**NPY1R**	4.61			4	**0.000010**		Vawter et al. (2004)
NRCAM			0.42	3	0.072400		Ayalew et al. (2012) and Roussos et al. (2012)
**NRG1**			0.24	6	**0.000002**		Carter (2009), Ayalew et al. (2012) and Berretta (2012)
**NTRK2**	3.99			6	**0.007300**	MA 3.34	Pandya, Kutiyanawalla & Pillai (2013)
PAX7			0.21	6	0.021400		
**PRKAB2**			0.27	5	**0.002002**		
**PRKAG3**			0.27	5	**0.002847**		
**SERPINA3**				(4)	**(0.0273)**	MA 0.26	Kim et al. (2016) and Tomasik et al. (2016)
**SLC2A4**			0.19	5	**0.001735**		
SLIT2	2.21			3	0.046500	MA 3.15	
TGFB1		1.25		13	0.397200	MA 2.15	Miller et al. (2011) and Berretta (2012)
**TIMP1**				(4)	(**0.0330**)	MA 2.87	Schwarz et al. (2010)
**TIMP2**				(4)	(**0.0257**)	MA 3.61	Kumarasinghe et al. (2013)
TNF	2.22			8	0.013100		Carter (2009) and Miller et al. (2011)

**Notes.**

Genes (symbols presented alphabetically) are listed when they were up- or down-regulated 2-fold or more in the medial rectus muscle samples from patients with exotropia. We list the fold-difference for the samples obtained from patients with exotropia compared with the normal controls, the number of independent samples (*n*), the *p*-value, and major references providing evidence when the gene is schizophrenia-related. We included values for two genes with unchanged expression, MMP9 and TGFB1, because they both are known to be relevant for muscle plasticity as well as schizophrenia. All genes that were significantly up- or down-regulated (below the adjusted *p*-value of ≤ 0.012500 according to Benjamini & Hochberg, 1995) are indicated in bold font (gene symbol and *p*-value); those that were near the adjusted *p*-value are shown in regular font. Genes that are schizophrenia-related (biomarkers or susceptibility genes) are indicated by gray shading. When available, confirmation by microarray (MA, Altick et al., 2012) is indicated. *p*-values and *n* are given in parentheses when expression data is exclusively from microarrays rather than from PCR arrays. All expression changes in the MA “Confirmation” column are statistically significant (Altick et al., 2012). Only genes encoding signaling molecules, but not structural muscle proteins (such as DES, DMD, DYSF, MUSK, NEB, TNNI2, TNNT3, TRIM63, TTN) are included, since muscle proteins involved in contractility were the focus of our previous publication (Agarwal et al., 2016). Abbreviations used: MA, microarray; *n*, number of independent experiments (pair-wise comparison of age-matched muscle samples).

### Thirteen of 28 dysregulated genes in lateral rectus muscles from patients with esotropia were schizophrenia-related

When we tested gene expression in lateral rectus muscles from patients with esotropia, we found that 28 genes encoding signaling molecules were significantly altered. Among these 28 genes, 13 were schizophrenia-related. Two of these genes, NRG1 and SERPINA3, were down-regulated, while 11 others were significantly up-regulated: BDNF, CTGF, IL1B, IL10RA, MMP2, NPY1R, NTRK2, TGFB1, TIMP1, TIMP2, and TNF (all statistically significant, with an adjusted *p* ≤ 0.018182). Three additional genes, GSK3B, MIF, and MMP9, were borderline, with *p*-values of 0.022137, 0.024364, and 0.022581, respectively. Although expression of PHOX2B (PMX2B/ARIX), which has been implicated in both strabismus and schizophrenia (Toyota et al., 2004), was reduced to 50% in the muscles from patients with strabismus, this reduction was not statistically significant in our data set (*p* = 0.13, *n* = 4). All genes with potentially significant alterations (based on original and adjusted *p*-values) are compiled in Table 4, using the same specifications as for Tables 2 and 3. These data indicate that more schizophrenia-related genes were significantly altered in expression in the lateral rectus muscles from patients with esotropia than in the medial rectus muscles from patients with exotropia.

**Table 4:** Altered expression of signaling molecules in *lateral* rectus muscles from patients with esotropia, with gray shading indicating schizophrenia-related genes. The fold-difference compared with normal (donor) lateral rectus muscles is shown. For gene names, see Table 1.

	Fold difference				
Gene symbol	Increase +	Decrease −	*n*	*p*-value	Reference for schizophrenia
**BDNF**	3.20		4	**0.000961**	Pandya, Kutiyanawalla & Pillai (2013)
**CCL16**	7.46		4	**0.003023**	
**CNTFR**	6.47		4	**0.000542**	
**CNTN1**	12.63		4	**0.004910**	
**CTGF**	5.11		4	**0.004689**	Schwarz et al. (2010)
**DCX**	8.24		4	**0.010925**	
**DDR2**	3.79		4	**0.000588**	
GDNF		0.05	4	0.029201	
GSK3B	1.69		4	0.022137	Jope & Roh (2006) and Emamian (2012)
**ICAM1**	2.35		4	**0.009534**	
**IL1A**	28.49		4	**0.003214**	
**IL1B**	13.81		4	**0.005604**	Xu & He (2010)
**IL10RA**	3.43		4	**0.000925**	Carter (2009)
MIF	1.75		4	0.024364	Schwarz et al. (2010)
**MMP2**	5.44		4	**0.000014**	Schwarz et al. (2010)
MMP9	4.61		4	0.022581	Kumarasinghe et al. (2013)
**NGF**	3.48		4	**0.014247**	
**NOTCH2**	4.90		4	**0.001907**	
**NPFFR2**	19.72		4	**0.003034**	
**NPY1R**	5.53		4	**0.000409**	Vawter et al. (2004)
**NRG1**		0.24	4	**0.010607**	Carter (2009), Ayalew et al. (2012) and Berretta (2012)
**NTRK1**	6.73		4	**0.000148**	
**NTRK2**	5.98		4	**0.001411**	Pandya, Kutiyanawalla & Pillai (2013)
**PAX7**		0.03	4	**0.014824**	
**PTGER2**	26.35		4	**0.000006**	
**SERPINA3**		0.25	4	**0.005237**	Kim et al. (2016) and Tomasik et al. (2016)
**SLIT2**	4.49		4	**0.010130**	
**SPP1**		0.25	4	**0.004008**	
STAT3	2.12		4	0.019094	
**TGFB1**	3.98		4	**0.016445**	Miller et al. (2011) and Berretta (2012)
**TIMP1**	6.69		4	**0.000001**	Schwarz et al. (2010)
**TIMP2**	7.05		4	**0.002814**	Kumarasinghe et al. (2013)
**TNF**	8.03		4	**0.000719**	Carter (2009) and Miller et al. (2011)

**Notes.**

Genes (symbols presented alphabetically) are listed when they were up- or down-regulated approximately 2-fold or more in the lateral rectus muscle samples from patients with esotropia. We list the fold-difference for the samples obtained from patients with esotropia compared with the normal controls, the number of independent samples (*n*), the *p*-value, and major references providing evidence when the gene is schizophrenia-related. All genes that were significantly up- or down-regulated (with an adjusted *p*-value of ≤ 0.018182 according to Benjamini & Hochberg, 1995) are indicated in bold font (gene symbol and *p*-value); those that were near the adjusted *p*-value are shown in regular font. Genes that are schizophrenia-related (biomarkers or susceptibility genes) are indicated by gray shading. Abbreviations used: *n*, number of independent experiments (pair-wise comparison of age-matched muscle samples).

### Interpretation of the medial vs. lateral rectus muscle gene expression data: what is normal, increased or reduced?

The finding that more schizophrenia-related genes with significantly altered expression levels were observed in the lateral rectus than medial rectus muscles suggested that schizophrenia-relatedness is not specific to the medial rectus muscle when the patient has developed a deviation. However, these results are not entirely conclusive, because the PCR arrays only inform about *relative* differences between two paired muscles, and we examined in these data sets specifically whether gene expression was altered between normal rectus muscles and rectus muscles from patients with strabismus, separately for each muscle type (medial and lateral rectus). If there were already differences in basal levels of gene expression between normal medial and normal lateral rectus muscles, then we would miss potentially important absolute differences in gene expression. Accordingly, to conclusively interpret our data, we tested in a separate series of experiments whether *normal* medial and lateral rectus muscles differ in gene expression levels, and we also determined whether *medial* rectus muscles from patients with exotropia showed alterations that differed from those found in *lateral* rectus muscles from patients with esotropia.

### Genes with significant differences in expression levels between normal horizontal rectus muscles

When we compared gene expression differences between normal medial and normal lateral rectus muscles for 76 signaling molecules, we found that only four genes had significantly lower levels of expression in normal medial than in normal lateral rectus muscles: NPY1R, NTRK2, SLIT2 and TIMP2 (adjusted *p* ≤ 0.021429, *n* = 4). With the exception of SLIT2, these genes are known to be schizophrenia-related (see references in Table 5). In addition, the schizophrenia-related genes IL10RA and MMP2 showed a trend towards a reduction, to 33% and 45%, respectively, in medial rectus muscles in this data set (Table 5). These data indicate that basal levels of expression of at least three schizophrenia-related genes are lower in normal medial rectus muscles than in normal lateral rectus muscles, independent of the strabismic condition.

**Table 5:** Signaling molecules with altered expression between normal medial (MR) and normal lateral rectus (LR) muscles, with gray shading indicating schizophrenia-related genes. For gene names, see Table 1.

	MR Fold Difference over LR			
Gene symbol	Decrease −	*n*	*p*-value	Reference for schizophrenia
IL10RA	0.33	4	0.0327	Carter (2009)
MMP2	0.45	4	0.0986	Schwarz et al. (2010)
NOTCH2	0.48	4	0.0317	
**NPY1R**	0.38	4	**0.0043**	Vawter et al. (2004)
**NTRK2**	0.37	4	**0.0045**	Pandya, Kutiyanawalla & Pillai (2013)
**SLIT2**	0.43	4	**0.0147**	
**TIMP2**	0.57	4	**0.0168**	Kumarasinghe et al. (2013)

**Notes.**

Genes (symbols listed alphabetically) are included when they had an approximately 2-fold or more altered level of expression in the normal medial rectus muscle as compared with the normal lateral rectus samples. All genes that were significantly up- or down-regulated (with an adjusted *p* value ≤ 0.021429 according to Benjamini & Hochberg, 1995) are indicated in bold font (gene symbol and *p*-value); those that were near the adjusted *p*-value are shown in regular font. We list the fold-difference for the medial rectus (MR) sample compared with the lateral rectus (LR), the number of independent samples (*n*), the *p*-value, and major references providing evidence when the gene is schizophrenia-related. Genes that are schizophrenia-related (biomarkers or susceptibility genes) are indicated by gray shading. Abbreviations used: *n*, number of independent experiments (pair-wise comparison of age-matched muscle samples).

**Table 6:** Altered expression of signaling molecules between lateral rectus (LR) muscles from patients with esotropia and medial rectus (MR) muscles from patients with exotropia, with gray shading indicating schizophrenia-related genes. The fold-difference of the MR muscles over the LR muscles is shown. For gene names, see Table 1.

	MR fold difference over LR					
Gene symbol	Increase +	Decrease −	*n*	*p*-value	Confirmation	Reference for schizophrenia
BDNF		0.38	5	0.024653		Pandya, Kutiyanawalla & Pillai (2013)
BMP4		0.52	5	0.010259		
CTGF		0.60	5	0.039498		Schwarz et al. (2010)
**DDR2**		0.38	5	**0.001261**		
GSK3B		0.67	5	0.032599		Jope & Roh (2006)
**IL1A**		0.16	5	**0.004172**		Emamian (2012) and Xu & He (2010)
IL1B		0.29	5	0.034609		Hänninen et al. (2008)
IL6R		0.48	5	0.009644		Miller et al. (2011)
MMP1		0.42	5	0.028093		Kim et al. (2012)
MMP2		0.44	5	0.010358	Kitada et al. (2003)	Schwarz et al. (2010)
**NGF**		0.44	5	**0.000180**		
NLGN1		0.47	3	0.062631		Zhang et al. (2015)
**NOTCH2**		0.43	5	**0.006422**		
**NTRK2**		0.40	5	**0.006887**		Pandya, Kutiyanawalla & Pillai (2013)
PAX3	1.62		5	0.041448		
**PTGER2**		0.23	5	**0.003739**		
RELN	1.75		3	0.057063		Berretta (2012) and Ovadia & Shifman (2011)
**TCF4**		0.52	3	**0.005527**		Stefansson et al. (2009) and Wirgenes et al. (2012)
TIMP2		0.50	5	0.038821	Kitada et al. (2003)	Kumarasinghe et al. (2013)

**Notes.**

Genes (symbols listed alphabetically) are included when they had an approximately 2-fold or more altered level of expression in the medial rectus muscle samples from patients with esotropia as compared with the lateral rectus samples obtained from patients with exotropia. All genes that were significantly up- or down-regulated (with an adjusted *p* value ≤ 0.007895 according to Benjamini & Hochberg, 1995) are indicated in bold font (gene symbol and *p*-value); those that were near the adjusted *p*-value are shown in regular font. We list the fold-difference for the medial rectus (MR) sample compared with the lateral rectus (LR), the number of independent samples (*n*), the *p*-value, and major references providing evidence when the gene is schizophrenia-related. Genes that are schizophrenia-related (biomarkers or susceptibility genes) are indicated by gray shading. When available, confirming evidence (reference) for the gene expression difference is given. Abbreviations used: *n*, number of independent experiments (pair-wise comparison of age-matched muscle samples).

### Differences in gene expression between horizontal rectus muscles from patients with strabismus by direct comparison

We next compared gene expression levels between medial rectus muscles from patients with exotropia and lateral rectus muscles from patients with esotropia for 100 genes encoding signaling molecules. Seven of the 100 genes tested here showed significant differences between the two muscles (adjusted *p* ≤ 0.007895). Of these seven genes, two are known to be schizophrenia-related: NTRK2 and TCF4 (which also regulates myogenesis, Mathew et al., 2011), and both showed a lower level of gene expression in the medial rectus compared with the lateral rectus muscle (Table 6). In addition, several schizophrenia-related genes showed a trend towards lower expression in the medial rectus muscle from patients with exotropia: BDNF, CTGF, GSK3B, IL1B, IL6R, MMP1, MMP2, NLGN1 and TIMP2. The schizophrenia- and strabismus-implicated PHOX2B gene (Toyota et al., 2004) was expressed at a lower level in medial rectus muscles than lateral rectus muscles, but the reduction (to 54%) was not statistically significant in our data set (*p* = 0.14, *n* = 5). The altered expression of at least two schizophrenia-related genes could point to a muscle-specific deficit of a subset of signaling molecules, resulting in increased vulnerability of the medial rectus as compared with the lateral rectus muscles. Some of the trends in gene expression differences between horizontal muscles from patients with strabismus, notably for MMP2 and TIMP2, were similar to those reported previously (Kitada et al., 2003).

**Figure 3:**
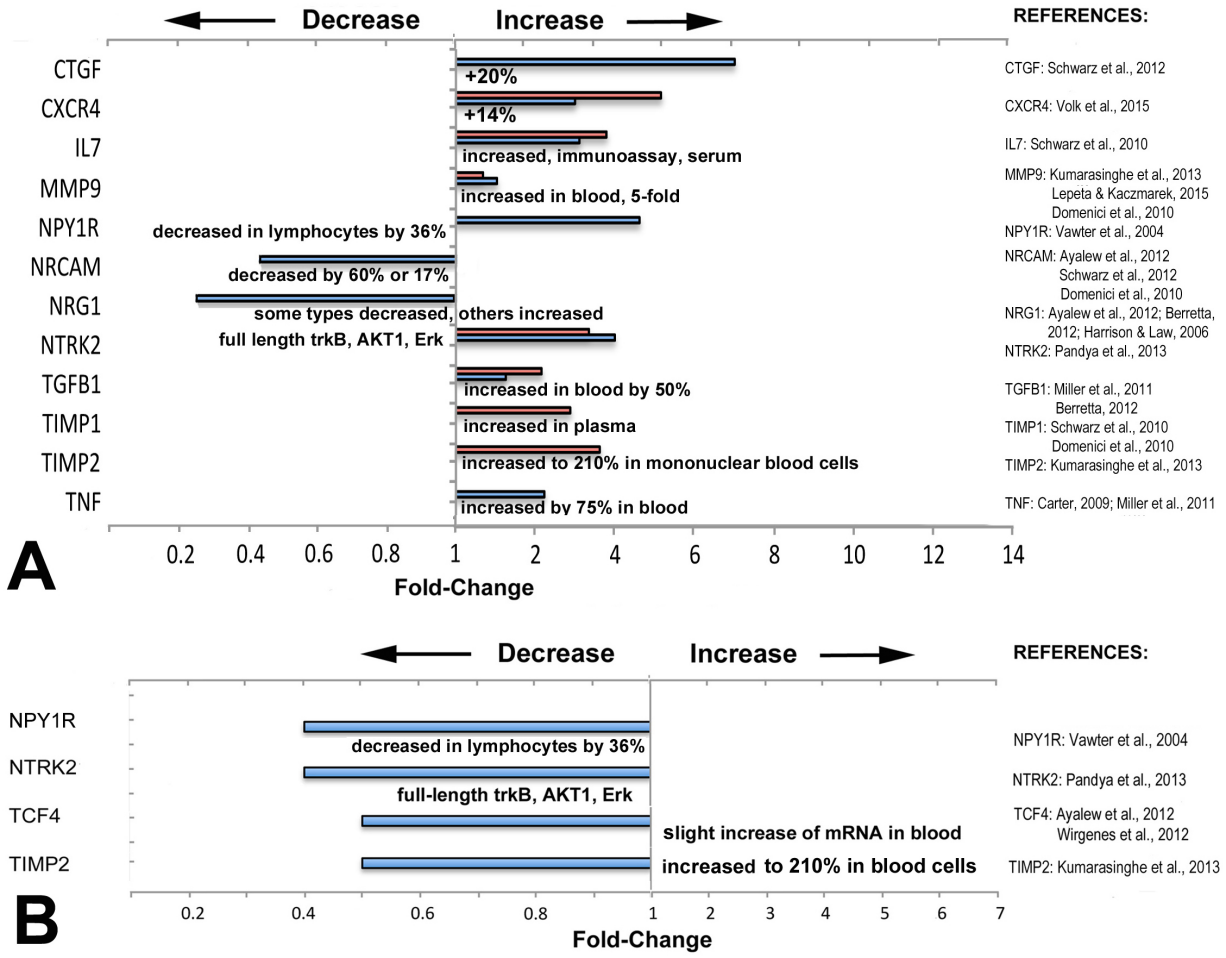
Directionality of altered expression of schizophrenia-related genes in extraocular muscles. (A) Directionality of altered expression of schizophrenia-related genes *when medial rectus muscles have become dysfunctional*, compared with tissues from schizophrenia patients (from the literature cited in Tables 2–6, and Domenici et al., 2010; Harrison & Law, 2006; Lepeta & Kaczmarek, 2015). Blue bars indicate data from PCR arrays, red bars indicate data from microarrays (Altick et al., 2012). References on the right contain the information about directionalities of changes of gene or protein expression in tissues from patients with schizophrenia. (B) Extent of apparent *absolute* deficits in expression of schizophrenia-related genes in medial rectus muscles from patients with exotropia (blue bars) as compared with the directionality of gene expression in tissues from schizophrenia patients (narrative, from the literature). Gene expression changes for medial rectus muscles compared with lateral rectus muscles.

### Most of the schizophrenia genes with altered expression in EOMs from patients with strabismus were altered in the same direction as in other tissues or fluids from schizophrenia patients

To determine whether the direction of expression (over- or under-expression) matches with the direction reported in previous studies on biomarkers in tissues from schizophrenia patients, we compared directionality and compiled the data in Fig. 3. The information for schizophrenia biomarkers and schizophrenia-relevant gene expression was obtained from studies that quantified proteins in blood or in cerebrospinal fluid (CSF), or examined gene expression in brain tissues from patients with schizophrenia, as listed in Fig. 3. We first compiled data separately to compare relative changes: those between normal rectus muscles from donors and muscles from patients with horizontal strabismus (Fig. 3A). For these comparisons, some data was derived from microarrays on predominantly medial rectus tissues from our previous report (Altick et al., 2012). We also compared the direction of the absolute changes of the baseline expression between normal medial and lateral rectus muscles, and between medial and lateral rectus muscles from patients with strabismus (Fig. 3B). As can be seen in the graphs, most schizophrenia-related genes, including those with trends rather than statistical significance, showed increased expression when patients had horizontal strabismus, which was similar to reports from the biomarker and susceptibility literature in schizophrenia (CTGF, CXCR4, IL7, MMP9, TGFB1, TIMP1, TIMP2, and TNF, Fig. 3A), while NRG1 was decreased in EOMs from patients with strabismus as well as in blood from patients with schizophrenia (Zhang et al., 2008; Wang et al., 2015). However, there were also examples where the directionality of relative changes was reversed, or was unclear (NPY1R, NTRK2, Fig. 3A). The data for inherent, absolute “baselines” of expression between medial and lateral rectus muscles revealed two genes with similar directionalities between EOMs and schizophrenia (NPYR1, NTRK2), while two others (TCF4, TIMP2) went in the opposite direction (slight increase in blood, Fig. 3B). Genes with consistently lower expression levels in both medial rectus and schizophrenia tissues included NPY1R, TIMP2, and possibly NTRK2. Overall, these comparisons do not reveal a pattern in changes of schizophrenia-related gene expression that supports specificity for medial rectus muscles from patients with exotropia.

## Discussion

Previous studies reported a surprisingly strong association between exotropia and schizophrenia (Korn, 2004; Toyota et al., 2004; Schiffman et al., 2006; Yoshitsugu et al., 2006; Ndlovu et al., 2011; Toyosima et al., 2011). There are two possibilities how exotropia may relate to schizophrenia. Exotropia may occur “in parallel” to the abnormal brain development and subsequent schizophrenia, as depicted in Fig. 2A. This could be due to imbalanced expression of signaling molecules which affect both, the regulation of extraocular muscles (EOMs), as well as regulation of early brain development. This would render exotropia an epiphenomenon, but possibly a useful predictor for schizophrenia (allowing earlier diagnosis and intervention). The alternative possibility is that exotropia may be serial to schizophrenia, possibly in a causal relationship, as depicted in Fig. 2B. This relationship, that exotropia or a convergence weakness may contribute to the cause of schizophrenia, has been hypothesized (Korn, 2004), and receives support through studies showing that people with congenital blindness or very early loss of vision are “immune” to developing schizophrenia (Landgraf & Osterheider, 2013; Silverstein, Wang & Keane, 2013). This suggests that abnormal visual development or abnormal visual experiences are essential in the pathogenesis of most, if not all, cases of schizophrenia. Consistent with this model (model B), the visual cortex of people with schizophrenia is structurally reduced (Dorph-Petersen et al., 2007). The second model implicates exotropia as a potential true risk factor, with a contributing causative role in the development of schizophrenia. Since exotropia can be treated (by corrective surgery), this model would have significant clinical implications, because correction of the exotropia at an early age or in a timely manner may normalize vision (Hainline & Riddell, 1996; Abroms et al., 2001; Paik & Yim, 2002; Von Noorden & Campos, 2002; Schubert, Henriksson & McNeil, 2005; Lennerstrand, 2007; Suh et al., 2013), and thereby may eliminate or reduce a risk factor for schizophrenia.

We here examined potential evidence for the first model (model A). If the same signaling molecules involved in schizophrenia also regulate eye alignment, and their expression is imbalanced in EOMs from patients with exotropia, and only exotropia, but not esotropia, is predictive for schizophrenia, then one would expect that the two eye muscles, medial rectus and lateral rectus, differ in the expression of those imbalanced signaling molecules. In our data, we did not find conclusive evidence that supports model A, and accordingly this indicates that model B should receive further scrutiny.

### Technical considerations and limitations of our approach

We measured gene expression in PCR arrays rather than in microarrays or by using proteomics, because PCR arrays proved to be more sensitive than the proteomics approach and more versatile and targeted than microarrays (Agarwal et al., 2016). It is important to consider limitations of our approach. These include sensitivity of our assay, restriction of muscle samples to the distal part of EOMs rather than whole EOMs, and the premise that differences in gene expression would be apparent, if present, between medial rectus muscles in exotropia and lateral rectus muscles in esotropia. Although we collected samples from muscles that appeared to have, with few exceptions, “normal” contractility by qualitative observation (see Table S1), we do not know the contractility status of the muscle’s antagonist that was not surgically resected. The fact that gene expression changes were rather consistent among strabismus cases suggests that gene expression changes in muscles from patients with strabismus indicate “dysfunction” (inability to align the eyes) rather than correlate bi-directionally with either overacting (hypertrophic) or underacting (hypotrophic) muscles—a classification that is now considered less informative (Kushner, 2010). According to a recent review (Nasrallah, 2013), there are 365 biomarkers for schizophrenia, so we could have missed some relevant candidates. It is possible that additional genes, not examined here, are differentially expressed. Nevertheless, we obtained information about gene expression levels for a large number of signaling molecules.

Conceivably, some of our *p*-values may have been significant with larger sample sizes. For this reason, we report trends when the values were close to the required *p*-value (adjusted *p*-values for multiple comparisons as indicated in the legends to Tables 2–6). The causes of strabismus are heterogeneous (Von Noorden & Campos, 2002; Kushner, 2010; Von Bartheld, Croes & Johnson, 2010; Ye et al., 2014), and such heterogeneity increases variability, although we sought to include only muscles from “common” idiopathic strabismus cases (mostly constant exotropia or esotropia) and exclude complicated forms with nystagmus, neurological etiology, or thyroid-associated ophthalmopathy. In cases of horizontal strabismus, the reason for the strabismus is not necessarily restricted to one of the two muscles, but one muscle type may be underacting, and the opposing one may be overacting. We had to restrict our analyses, for practical reasons, to the medial rectus in exotropia, and the lateral rectus in esotropia, because the weakened (recessed) muscle generates little, if any tissue for gene expression analysis. Accordingly, if gene expression changes affected primarily or exclusively the opposing (not resected) muscle type rather than the resected muscle type, we would have missed this in our approach. However, based on recent findings implicating the agonist as a major contributor to the deviation in horizontal strabismus (Debert et al., 2016), we consider it unlikely that the muscle type operated upon would not show any relevant gene expression changes.

### Interpretation of our results

We identified a surprisingly large number of schizophrenia-related signaling molecules that are dysregulated in EOMs from patients with strabismus (Table 2). Initially, this seemed to be a possible explanation for the exotropia specificity (Christensen et al., 2015; Von Bartheld et al., 2016). However, when we examined gene expression separately in medial rectus and lateral rectus muscles derived from patients with exotropia and esotropia, respectively, it became apparent that the changes in expression of “schizophrenia genes” were not specific to the medial rectus muscles. The lack of specificity and the larger number of dysregulated schizophrenia genes in lateral rectus muscles do not favor a muscle-specific vulnerability of the medial rectus muscle, and accordingly do not support model A (Fig. 2A).

**Figure 4:**
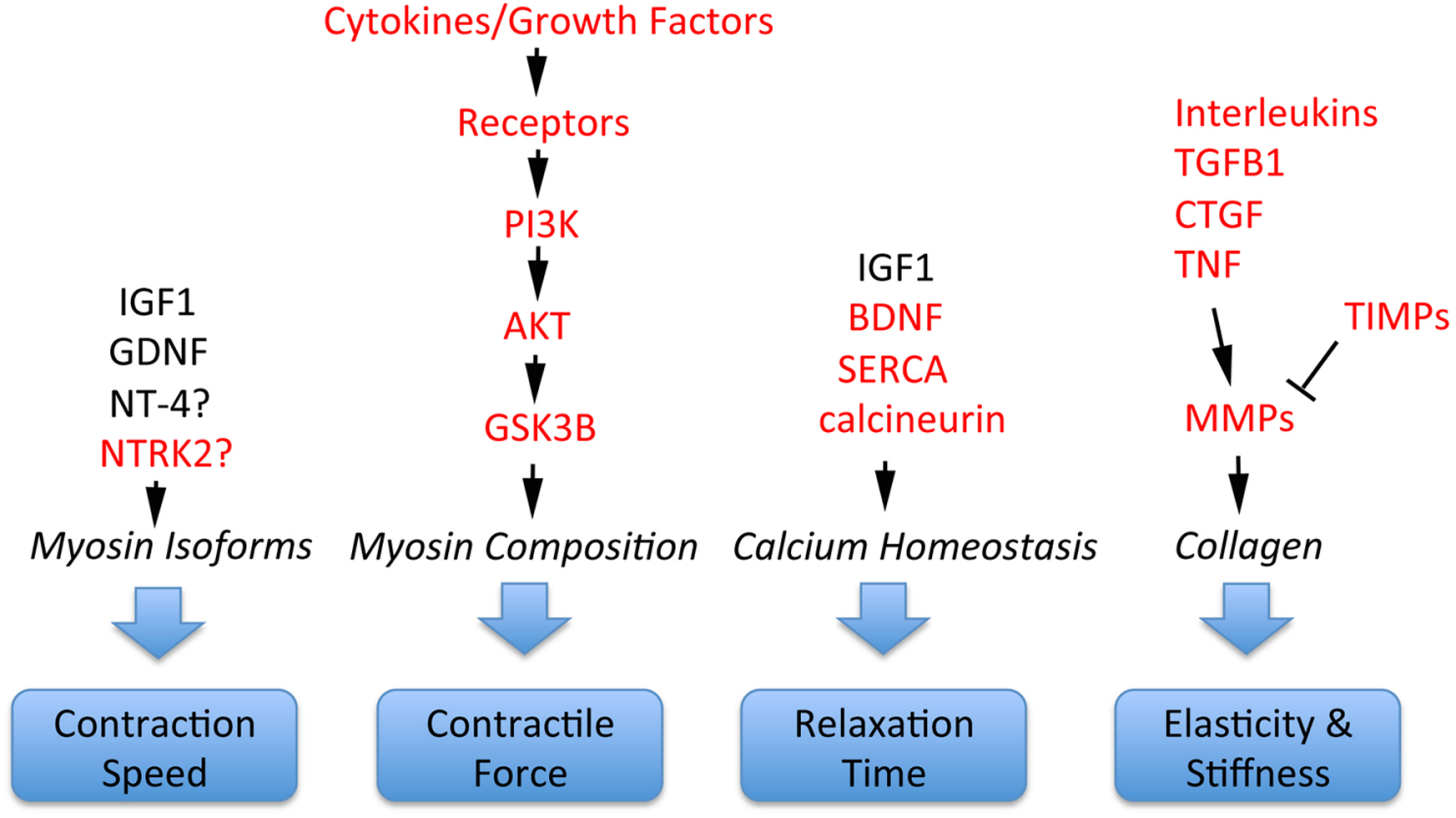
Involvement of schizophrenia-related genes and gene products (red font) within signaling pathways that regulate key functions of extraocular muscle development and plasticity. The indicated genes and gene products are important for generating superfast contractions (saccades), adjusting muscle force, relaxation time, and the elastic properties essential for eye movements. For details, see Kjaer (2004), Li, Feng & Von Bartheld (2011), Stager Jr, McLoon & Felius (2013), Mienaltowski & Birk (2014), Agarwal et al. (2016) and Nelson, Stevens & McLoon (2016).

Schizophrenia-related genes and proteins are involved in the regulation of multiple important EOM properties. These include myosin heavy chain composition for adjustment of contractile properties; contraction speed to execute effective saccades; control of relaxation time; and elasticity/stiffness (altered collagen composition), as illustrated in Fig. 4. All of these properties are regulated by signaling pathways where schizophrenia-related genes or gene products have key functions. For example, contractile properties are controlled by cytokines and growth factors that signal through PI3K, AKT and GSK3B pathways to affect myosin heavy chain composition of myofibers (Kjaer, 2004; Li, Feng & Von Bartheld, 2011); contraction speed and relaxation time are regulated by EOM-derived growth factors such as GDNF and the neurotrophins BDNF and NT-4 (Chen & Von Bartheld, 2004; Von Bartheld, Croes & Johnson, 2010; Li, Feng & Von Bartheld, 2011; Willoughby et al., 2015; Nelson, Stevens & McLoon, 2016); relaxation time is also controlled by calcium handling through SERCA/DISC1 (Li, Feng & Von Bartheld, 2011; Nelson, Stevens & McLoon, 2016), molecules that are implicated in abnormal calcium homeostasis in schizophrenia (Park et al., 2015). Furthermore, the important properties of elasticity and EOM stiffness are controlled by growth factors and cytokines (CTGF, TGFB, TNF, interleukins) that, together with TIMPs, control via MMPs the turnover and alteration of collagen (Metcalfe & Weetman, 1994; Kjaer, 2004; Mienaltowski & Birk, 2014; Agarwal et al., 2016), as summarized in Fig. 4. Additional interactions between these components are the presentation of growth factors through the extracellular matrix, and the conversion of some neurotrophic factors from pro-forms to mature forms, with functional consequences (Pandya, Kutiyanawalla & Pillai, 2013).

Thus, a surprisingly large number of signaling molecules and pathways has been implicated in both EOM function/plasticity, as described above, as well as in schizophrenia pathogenesis (Carter, 2009; Schwarz et al., 2010; Miller et al., 2011; Kim et al., 2012; Kumarasinghe et al., 2013; Pandya, Kutiyanawalla & Pillai, 2013; Chopra, Baveja & Kuhad, 2015). While utilization of the same signaling pathways in the two disorders may be a coincidence, it is also possible that some genes may be schizophrenia-associated, simply because they facilitate exotropia, which in turn may lead—via abnormal early visual experiences—to schizophrenia (Fig. 2B, Schubert, Henriksson & McNeil, 2005). It has been known for nearly a century that eye movement abnormalities in smooth pursuit and saccades have the highest frequency (∼80%) among co-morbidities in schizophrenia (Levy et al., 1993), but the role of exotropia was recognized only recently. Exotropia may be fundamental to a wide range of visual abnormalities affecting visual cortex, saccades and smooth pursuit eye movements, as part of the broader schizophrenia syndrome. The specificity of schizophrenia’s association with exotropia, but not with any other childhood vision problems, may lie in the tendency for exotropia to develop intermittently before the deviation becomes constant (Von Noorden & Campos, 2002, page 249). This would allow preservation of vision in both eyes beyond the critical period (unlike esotropia which more often leads to amblyopia). Different percepts of realities conveyed through both eyes in exotropia (Economides, Adams & Horton, 2012) thus may facilitate the morphing and tolerance of multiple realities (Korn, 2004).

Overall, our study identified 16 genes with potential roles in both strabismus and schizophrenia (shaded in Tables 2–6); eight additional genes showed trends, but missed the *p*-value for significance. Interestingly, many of these genes (MMP2, NPY1R, NTRK2, CTGF, TIMP1, TIMP2, MMP2, TNF) are involved in tissue remodeling, including composition of muscle and tendon, remodeling of extracellular matrix and collagen, satellite cell migration and differentiation, as well as angiogenesis, as discussed in our previous report (Agarwal et al., 2016). Could these genes alone be responsible for exotropia’s increased odds ratio to develop schizophrenia? Most schizophrenia-related genes, individually, are thought to increase the odds of developing schizophrenia only very little—they typically have an odds ratio of less than 1.3 (Stefansson et al., 2009; Mulle, 2012). It has been proposed that schizophrenia-risk factors combine, and multiple risk factors together may add up to a more significant risk (Gottesman, 1991; Hänninen et al., 2008; Toyosima et al., 2011). When a certain threshold is reached, then schizophrenia may develop. Likewise, the more common forms of strabismus are thought to be the result of interactions between several susceptibility genes (Engle, 2007; Mohney et al., 2008). Accordingly, if dysregulation (expression at a higher or lower level than normal) of several schizophrenia-related and/or strabismus-related genes are combined, then a certain threshold may be reached, strabismus may result, and likewise the patient may develop schizophrenia.

### Epidemiology and clinical implications

The following epidemiological considerations underscore the magnitude and the clinical implications of the exotropia-schizophrenia link. The prevalence of schizophrenia worldwide is estimated at 0.5–0.7%, or about 45 million people (Saha et al., 2005; Messias, Chen & Eaton, 2007; McGrath et al., 2008). The prevalence of exotropia has been estimated to be 1.25% (Chew et al., 1994). Applied to the current world population, this amounts to 94 million people, with nearly 1/3 of them having constant exotropia at 3 years of age (Matsuo et al., 2007). According to published tables (Toyota et al., 2004; Yoshitsugu et al., 2006; Ndlovu et al., 2011), the odds ratio to develop schizophrenia is 39 (range: 14–85) for constant exotropia, and it is 2.3 (range: 1.7–3.2) for intermittent exotropia. With a neutral odds ratio (0.6%), 0.186 million people of the 31 million people with constant exotropia would develop schizophrenia. With an odds ratio of 39, this number is increased to 7.25 million. This means that among the 94 million people worldwide with exotropia (intermittent and constant), about 8.1 million (nearly 10%) will develop schizophrenia. Regardless of the exact odds ratio, it is obvious that a substantial percentage of people with schizophrenia have exotropia as a potentially co-associated factor. Exotropia has been increasing over esotropia in Asian countries (Yu et al., 2002; Green-Simms & Mohney, 2010). Consistent with an increase in exotropia in Asian countries, the prevalence of schizophrenia has nearly doubled in China from 1990 to 2010 (Chan et al., 2015), while it has been relatively stable in the USA and the UK (Cowan et al., 2011; Kirkbride et al., 2012) where the prevalence of exotropia did not change. Previous work examined whether timing of corrective surgery has an effect on later development of mental illness (Kilgore et al., 2014), but in the cases examined in that study, surgery was performed on children with intermittent (not constant) exotropia, the “early” surgery (at 3–6 years of age) may have been too late, and the patients were evaluated for mental illness only until early adulthood (up to a median age of 21.8 years), when schizophrenia would mostly not yet have been apparent, and therefore would have underestimated the occurrence of this type of mental disease. Future retrospective research will be needed to determine whether infants with constant exotropia who have early or timely corrective surgery (Abroms et al., 2001; Paik & Yim, 2002; Suh et al., 2013) develop schizophrenia less often than those that had corrective surgery later or had no corrective surgery at all. It will also be important to identify the types of exotropia that predominate among people with schizophrenia, and explore whether both, the time of onset and the duration of the exotropia, are important parameters not only for sensory and motor outcomes after surgery (Abroms et al., 2001; Hunter et al., 2001), but also for development of schizophrenia. The link between exotropia and schizophrenia has been much neglected and is understudied, despite the considerable potential for preventative interventions that requires more effective communication and collaboration between ophthalmologists and psychiatrists.

## Conclusions

Despite our findings of a surprisingly large number of schizophrenia-related genes with altered gene expression levels in eye muscles from people with strabismus, the lack of specificity for medial rectus muscles undermines a model of shared, region-specific gene expression abnormalities between exotropia and schizophrenia. Therefore, our data rather suggest consideration of the alternative model for the link between exotropia and schizophrenia, namely that exotropia-induced aberrant early visual experiences may enable and/or contribute as a causative factor to the development of schizophrenia.

##  Supplemental Information

10.7717/peerj.4214/supp-1 Table S1 Demographic Information for Samples used in PCR Arrays^1^ ^1^ Due to concerns about patient confidentiality and privacy, specified in four different IRB protocols and ethic boards over 16 years of sample collection, demographic and other patient information could not be collected for all subjects enrolled.^2^ Most of the cases were exotropias developed in infancy which were initially intermittent and then became constant or manifested very frequently (>50% of the time). Often, a medial rectus resection was done as a secondary procedure when a patient had residual (under-correction) or recurrent exotropia after alateral rectus recession had been done as the initial procedure.^3^ Assessment of the muscle’s contractility was based on how “tight” the muscle was when captured on a muscle hook to expose it.

10.7717/peerj.4214/supp-2 Table S2 Demographic Information, Entire Collection of Muscle Samples^1^ ^1^ Due to concerns about patient confidentiality and privacy, specified in four different IRB protocols and ethic boards over the 15 years of sample collection, demographic and other patient information could not be collected for all subjects enrolled. “Entire collection” includes samples used for microarrays and PCR arrays, as well as yet unused samples—but informs about the type of cases available over the past 15 years. ^2^ Most of the cases were exotropias developed in infancy which were initially intermittent and then became constant or manifested very frequently (>50% of the time). Often, a medial rectus resection was done as a secondary procedure when a patient had residual (under-correction) or recurrent exotropia after alateral rectus recession had been done as the initial procedure.^3^ Assessment of the muscle’s contractility was based on how “tight” the muscle was when captured on a muscle hook to expose it.

10.7717/peerj.4214/supp-3 Table S3 List of samples paired by age, gender, type for PCR arrays

10.7717/peerj.4214/supp-4 Table S4 Strabismic Medial Rectus (MR) and Lateral Rectus (LR) vs. donor data combined

10.7717/peerj.4214/supp-5 Table S5 Strabismic medial rectus (MR) vs. donor MR data

10.7717/peerj.4214/supp-6 Table S6 Strabismic lateral rectus (LR) vs donor LR data

10.7717/peerj.4214/supp-7 Table S7 Donor medial rectus (MR) vs donor lateral rectus (LR) data

10.7717/peerj.4214/supp-8 Table S8 Strabismic lateral rectus (LR) vs. strabismic medial rectus (MR) data

## Abbreviations

CSF: cerebrospinal fluid
EOM: extraocular muscle
LR: lateral rectus muscle
MA: microarray
MR: medial rectus muscle
PI3K: phosphoinositide 3-kinase
SERCA: sarcoplasmic/endoplasmic reticulum calcium ATPase; for abbreviations of genes, see Table 1

## Additional Information and Declarations

### Data Availability

The following information was supplied regarding data availability: The raw data of our PCR arrays are provided in Tables S4–S8.
