# Supplementary material for: Expression of schizophrenia biomarkers in extraocular muscles from patients with strabismus: an explanation for the link between exotropia and schizophrenia?
Source: PeerJ. 2017 Dec 22;5:e4214. doi: 10.7717/peerj.4214 (PMC5742522; doi:10.7717/peerj.4214)
Supplement: Table S1 — 1 Due to concerns about patient confidentiality and privacy, specified in four different IRB protocols and ethic boards over 16 years of sample collection, demographic and other patient information could not be collected for all subjects enrolled.2 Most of the cases were exotropias developed in infancy which were initially intermittent and then became constant or manifested very frequently (>50% of the time). Often, a medial rectus resection was done as a secondary procedure when a patient had residual (under-correction) or recurrent exotropia after alateral rectus recession had been done as the initial procedure.3 Assessment of the muscle’s contractility was based on how “tight” the muscle was when captured on a muscle hook to expose it. [file peerj-05-4214-s001.docx]

**Supplementary Table 1: Demographic Information for Samples used in PCR Arrays^1^**

**Diagnosis Esotropia Exotropia^2^**

**Age** (years)

Mean 24.8 22.1

Range 2–80 2–60

**Gender**

Male 12 9

Female 15 9

Unknown 1 0

**Ethnicity**

Caucasian 19 7

Hispanic 1 1

Asian 2 5

Unknown 6 5

**Duration of Misalignment** (when known)

Since infancy 11 5

**Surgical Procedure** (when known)

Lateral Rectus Resection 14

Lateral Rectus Recession 4 5

Medial Rectus Resection 6

Medial Rectus Recession 2

**Contractility of muscles^3^**

Normal 25 18

Increased 3 0

^1^ Due to concerns about patient confidentiality and privacy, specified in four different IRB protocols and ethic boards over 16 years of sample collection, demographic and other patient information could not be collected for all subjects enrolled.

^2^ Most of the cases were exotropias developed in infancy which were initially intermittent and then became constant or manifested very frequently (>50% of the time). Often, a medial rectus resection was done as a secondary procedure when a patient had residual (under-correction) or recurrent exotropia after a lateral rectus recession had been done as the initial procedure.

^3^ Assessment of the muscle’s contractility was based on how “tight” the muscle was when captured on a muscle hook to expose it.
