# Supplementary material for: Expression of schizophrenia biomarkers in extraocular muscles from patients with strabismus: an explanation for the link between exotropia and schizophrenia?
Source: PeerJ. 2017 Dec 22;5:e4214. doi: 10.7717/peerj.4214 (PMC5742522; doi:10.7717/peerj.4214)
Supplement: Table S2 — 1 Due to concerns about patient confidentiality and privacy, specified in four different IRB protocols and ethic boards over the 15 years of sample collection, demographic and other patient information could not be collected for all subjects enrolled. “Entire collection” includes samples used for microarrays and PCR arrays, as well as yet unused samples—but informs about the type of cases available over the past 15 years. 2 Most of the cases were exotropias developed in infancy which were initially intermittent and then became constant or manifested very frequently (>50% of the time). Often, a medial rectus resection was done as a secondary procedure when a patient had residual (under-correction) or recurrent exotropia after alateral rectus recession had been done as the initial procedure.3 Assessment of the muscle’s contractility was based on how “tight” the muscle was when captured on a muscle hook to expose it. [file peerj-05-4214-s002.docx]

**Supplementary Table 2: Demographic Information, Entire Collection of Muscle Samples^1^**

**Diagnosis Esotropia Exotropia^2^**

**Age** (years)

Mean 21.9 24.0

Range 1–81 2–80

**Gender**

Male 30 33

Female 43 38

Unknown 8 2

**Ethnicity**

Caucasian 42 24

Hispanic 1 6

Asian 14 26

Native American 0 1

Unknown 24 16

**Contractility of muscles^3^**

Normal 72 60

Increased 5 10

Decreased 3 0

Unknown 1 3

^1^ Due to concerns about patient confidentiality and privacy, specified in four different IRB protocols and ethic boards over the 15 years of sample collection, demographic and other patient information could not be collected for all subjects enrolled. “Entire collection” includes samples used for microarrays and PCR arrays, as well as yet unused samples – but informs about the type of cases available over the past 15 years.

^2^ Most of the cases were exotropias developed in infancy which were initially intermittent and then became constant or manifested very frequently (>50% of the time). Often, a medial rectus resection was done as a secondary procedure when a patient had residual (under-correction) or recurrent exotropia after a lateral rectus recession had been done as the initial procedure.

^3^ Assessment of the muscle’s contractility was based on how “tight” the muscle was when captured on a muscle hook to expose it.
